# A Promising Nano-Insulating-Oil for Industrial Application: Electrical Properties and Modification Mechanism

**DOI:** 10.3390/nano9050788

**Published:** 2019-05-23

**Authors:** Jiaqi Chen, Potao Sun, Wenxia Sima, Qianqiu Shao, Lian Ye, Chuang Li

**Affiliations:** State Key Laboratory of Power Transmission Equipment & System Security and New Technology, Chongqing University, Chongqing 400030, China; jqch@cqu.edu.cn (J.C.); sunpotao@cqu.edu.cn (P.S.); shaoqianqiu@cqu.edu.cn (Q.S.); yelian@cqu.edu.cn (L.Y.); lichuang123cqu@163.com (C.L.)

**Keywords:** C_60_ nanoparticles, electrical properties, insulating oil, modification mechanism, thermally stimulated current (TSC)

## Abstract

Despite being discovered more than 20 years ago, nanofluids still cannot be used in the power industry. The fundamental reason is that nano-insulating oil has poor stability, and its electrical performance decreases under negative impulse voltage. We found that C_60_ nanoparticles can maintain long-term stability in insulating oil without surface modification. C_60_ has strong electronegativity and photon absorption ability, which can comprehensively improve the electrical performance of insulating oil. This finding has great significance for the industrial application of nano-insulating oil. In this study, six concentrations of nano-C_60_ modified insulating oil (CMIO) were prepared, and their breakdown strength and dielectric properties were tested. The thermally stimulated current (TSC) curves of fresh oil (FO) and CMIO were experimentally determined. The test results indicate that C_60_ nanoparticles can simultaneously improve the positive and negative lightning impulse and power frequency breakdown voltage of insulating oil, while hardly increasing dielectric loss. At 150 mg/L, the positive and negative lightning impulse breakdown voltages of CMIO increased by 7.51% and 8.33%, respectively, compared with those of FO. The AC average breakdown voltage reached its peak (18.0% higher compared with FO) at a CMIO concentration of 200 mg/L. Based on the test results and the special properties of C_60_, we believe that changes in the trap parameters, the strong electron capture ability of C_60_, and the absorption capacity of C_60_ for photons enhanced the breakdown performance of insulating oil by C_60_ nanoparticles.

## 1. Introduction

Numerous studies on nano-insulating oil have been conducted since Argonne Laboratory proposed the concept of nanofluids in 1995 [1]. Thus far, no nano-insulating oil that can be used in the power industry has been discovered owing to the constraints caused by the following factors.

### 1.1. Long-Term Stability

Conventional nanoparticles (such as Fe_3_O_4_, TiO_2_, and Al_2_O_3_) are insoluble in oil. Matching surface modifiers must be used to ensure the long-term stability of these nanoparticles [2,3,4]. However, surface modifiers may deteriorate and fall off in long-term complex environments (such as heat, electricity, and magnetism), which weaken the dispersion of nanoparticles. Moreover, an improper amount of surface modifier may adversely affect the viscosity, as well as the physical and chemical stability, of the modified oil and even the heat transfer performance of nanoparticles [4].

### 1.2. Decrease in Negative Impulse Breakdown Performance

The positive impulse breakdown performance of most nano-insulating oils obviously improves. However, their negative impulse breakdown performance decreases to varying degrees, as shown in Table 1.

More than 90% of the lightning that occurs in nature is of negative polarity [13], therefore the decline in the negative impulse breakdown performance of nano-insulating oil restricts its application in power transformers [14].

### 1.3. Significant Increase in Dielectric Loss

The improvement of electrical properties of nano-insulating oil may be accompanied by a substantial increase in dielectric loss. This increase affects the heat dissipation of the transformer and accelerates the thermal aging of the dielectric.

Table 2 shows the results of the electrical performance test performed by Sartoratto [15] on Fe_3_O_4_ nanoparticle-modified insulating oil. The table shows that Fe_3_O_4_ nanoparticles (average nanoparticle size is 7.4 nm) increase the breakdown voltage of insulating oil and induce a significant increase in dielectric loss. Liu [12] and Mergos [16] have also observed this phenomenon in insulating oils modified by other nanoparticles such as ZnO, TiO_2_, and CuO.

Recently, we have found that nano-insulating oil made of C_60_ nanoparticles can greatly overcome these three problems due to its excellent properties. Theoretical calculations show that the electron affinity potential of C_60_ is approximately 2.683 eV [17], which is higher than that of the excellent gas-insulating medium SF_6_ (electron affinity is approximately 1.06 eV [18]). Therefore, C_60_ has a strong adsorption capacity for electrons. A single molecule can accept at least six electrons [19]. Moreover, C_60_ can absorb a large number of photons through photopolymerization [20]. The C_60_ molecule is soluble in organic solvents, especially aromatic solvents with large π bonds [21,22]. Therefore, C_60_ nanoparticles have a certain natural solubility in transformer insulating oils (the main component contains aromatic hydrocarbons) that are nonpolar solvents.

In this study, a new type of nanofluid was prepared by dispersing C_60_ nanoparticles into insulating oil without the use of a surface modifier. The breakdown strength and dielectric properties of the prepared samples were tested, and the thermally stimulated current (TSC) curves of fresh oil (FO) and nano-C_60_ modified insulating oil (CMIO) were experimentally determined. We discuss the mechanism involved in the enhancement of the breakdown performance of insulating oil on this basis.

## 2. Experimental Setup

### 2.1. Preparation of Nanofluids

Transformer mineral oil (#25) and 99.9% pure C_60_ powder (Tanfeng Tech, Suzhou, China) were used in the tests. The technical specifications of the mineral oil satisfied the international standard ASTM D3487-2000(II) [23].

The surface modifier may adversely affect oil material stability and heat transfer performance. Given that C_60_ particles have a certain solubility in insulating oil, surface modifiers were not used in the experiments. The photopolymerization of C_60_ can be prevented by protecting CMIO from light during preparation [24].

Pretreatments were carried out on all experimental samples as follows: First, mineral oil (#25) was filtered through a membrane filter for the removal of contaminants (the quality of the oil meets the requirement of CIGRE working group 12.17: particle content with a diameter larger than 5 μm is lower than 300 per 100 mL). Then, C_60_ particles and insulating oil were separately dried at 90 °C for 48 h in a vacuum drying oven. Finally, the mechanical grinding of the C_60_ nanoparticles and vacuum degassing of the insulating oil were performed.

Ultrasonication can effectively break the agglomeration and reduce particle size in fluid [25]. After the pretreatments, the C_60_ nanoparticles were placed in the insulating oil and ultrasonically dispersed at 60 °C for at least 60 min (sx-sonic FS-1800N, maximum volume =3 L, ultrasonic peak output = 1800 W, frequency = 20 kHz, ultrasonic horn diameter = 20 mm). The ultrasonic power used in the test was 70% of the maximum output power. After 6 seconds of continuous ultrasonic operation, it stopped for 6 seconds and then started the next operation (instrument setting) to prevent the ultrasonic horn and sample temperature from being too high. The applied energy per volume (weight) of nanoparticle material in suspension was estimated to be ca. 37.8 kJ per 50 mL of particles (0.015 g). The kinematic viscosity of the oil sample was approximately 10.8 mm^2^/s (40 °C). Mechanical agitation was required for 5 min after each 15 min of ultrasonication. CMIO samples with concentrations of 50, 100, 150, 200, 250, and 300 mg/L were prepared and dried in a vacuum oven for 48 h at 60 °C.

### 2.2. AC Breakdown Tests

**Figure 1:**
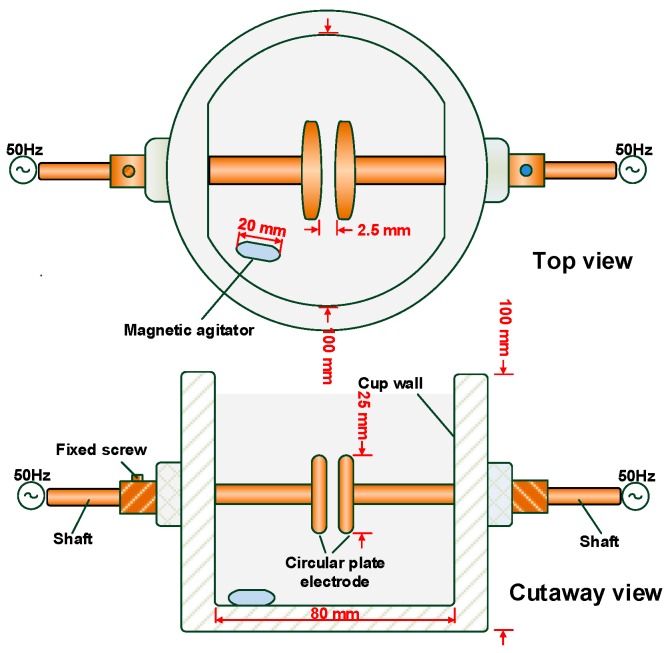
Sketch of the standard AC breakdown oil cup.

A Portable Oil Tester IJJD-80 (Ruixin Electric, Wuhan, China) (Figure 1) was used to measure AC breakdown voltage according to IEC-60156 [26]. The gap between the brass electrodes was set at 2.5 mm. A 50 Hz AC voltage with a rising rate of 2 kV/s was applied to the oil cup. The initial standing time was set at 5 min before the application of voltage. The time interval with stirring action and standing time after each breakdown were set at 2 min. Each sample was tested six times, and the average value was taken as the power-frequency breakdown voltage value. All the experiments were performed at room temperature.

### 2.3. Lightning Impulse Breakdown Tests

**Figure 2:**
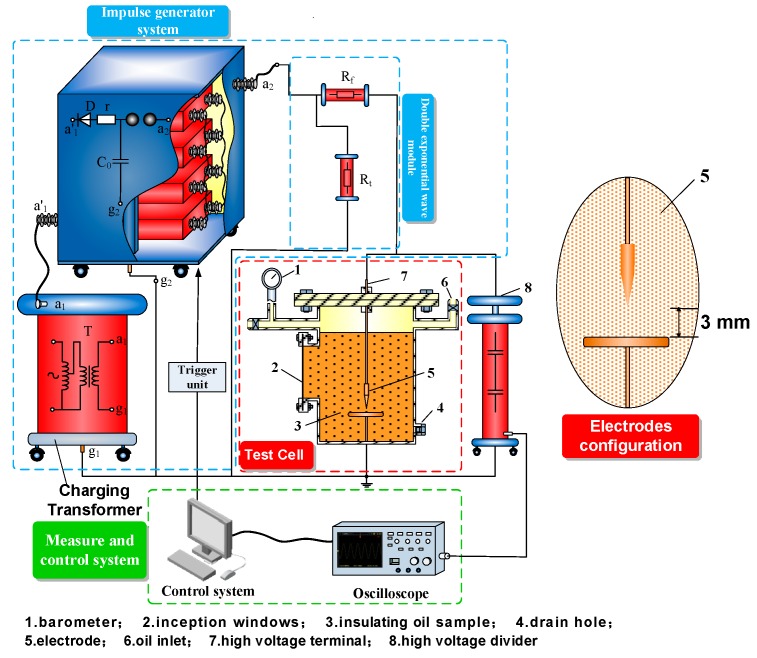
Sketch of the impulse voltage test platform.

A consecutive impulse generator (30 kJ/400 kV) was used, and voltage signals measured by a high voltage divider were recorded by a TDS2024C impulse analysis system (Xinyuan Electric, Yangzhou, China), as shown in Figure 2.

The 50% lightning breakdown voltages (hereinafter referred to as U_50_) of the FO and CMIO under positive and negative lightning impulse voltages were obtained through the up-and-down method [27]. Needle-plate electrodes with an oil gap of 3 mm were used, and the wavefront/tail time of the impulse voltage was 1.2/50 μs. The expected breakdown voltages of the positive and negative impulse breakdown voltages were set at 35 and 46 kV, respectively. Applied voltage varies in 1 kV of the expected breakdown voltage, and at least 50 effective shots in total were tested for each sample. The relaxation time between the consecutive shots was 5 min, and the agitation operation lasted for 1 min.

### 2.4. Frequency Domain Dielectric Spectrum Tests

A Novocontrol Concept 80 Broadband Dielectric Spectrometer (Novocontrol Tech, Montabaur, Germany) was used to measure the dielectric parameters of the FO and CMIO samples. The test frequency in this study ranged from 10^−2^ Hz to 10^7^ Hz, and the test temperature was set at 30 °C. The temperature accuracy was ±0.01 °C. A calibrating conversion module was adopted to prevent measurement error caused by leakage current.

### 2.5. TSC Tests

**Figure 3:**
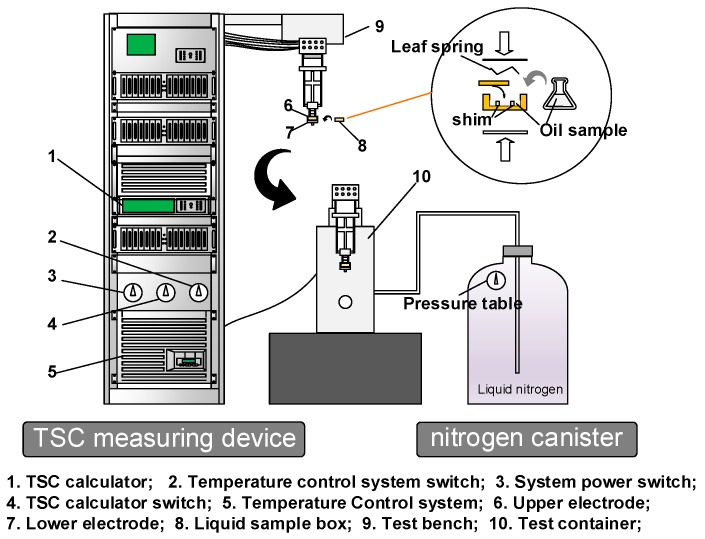
Thermally stimulated current (TSC) measurement system.

The trapping parameters of electrons in dielectric were determined through the TSC technique [28]. The curve plotting the dependence of TSC on temperature can be used in the calculation of trap level and trap density [29]. Figure 3 shows the TSC measurement system we used.

During the test, the temperature was adjusted with liquid nitrogen. Data were recorded using LabVIEW software (WinTSC V1.48). 

First, the oil sample was added to the sample cell, and a negative DC field of 4 kV/mm was applied to the tested sample for 30 min at 323 K. Then, the temperature was lowered to 243 K at a rate of 10 K/min for 15 min. Finally, the sample was heated to 353 K at a rate of 2 K/min. The current and sample temperature during the process were recorded.

### 2.6. Transmission Electron Microscopy (TEM)

C_60_ nanoparticles were examined with a high-resolution transmission electron microscope (HT7700, Hitachi, Japan). The maximum resolution of TEM used in the test was 0.204 nm, and the acceleration voltage was 100 kV.

### 2.7. Particle Size Distribution and Zeta Potential Tests

A Malvern Zetasizer Nano ZS90 (Malvern, UK) was used to measure the particle size distribution and zeta potential of the samples. The maximum particle size that the instrument can measure ranges from 5 μm to 0.3 nm, and the minimum sample volume for particle size distribution and zeta potential are only 20 and 150 μL, respectively.

## 3. Results

### 3.1. Zeta Potential and Particle Size Distribution of CMIO

Electrostatic repulsion among nanoparticles increases at high zeta potential, and this effect signifies good suspension stability [30,31]. Zeta potential test results are related to the pH and concentration of nanofluids [32,33]. Therefore, for the dispersed particles in insulating oil at 100 mg/L concentration, measurements were performed with a 633 nm He/Ne laser at a temperature of 25 °C according to the actual test requirements, so that the requirement of transmittance intensity was satisfied. The refractive index (RI) of C_60_ particles and insulating oil were set to 1.96 [34] and 1.48, respectively. We selected the test conditions as pH=7, and a total of 12 measurements were taken. The results are shown in Table 3.

An absolute value of zeta potential greater than 30 mV indicates good dispersion stability [31,35]. Therefore, the CMIO has good dispersion stability.

**Figure 4:**
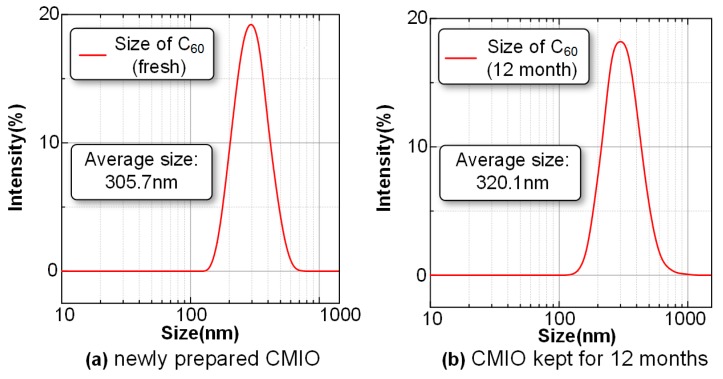
Particle size distribution of nano-C_60_ particles in CMIO. (**a**) newly prepared CMIO, (**b**) CMIO kept for 12 months. (Please note that whether a particle larger than 100 nm can be called a nanoparticle is still controversial.).

The particle size distribution of the newly prepared CMIO and the CMIO kept for 12 months were tested. The test results are shown in Figure 4.

Figure 4 shows that the particle size distribution of the C_60_ particles in the newly prepared CMIO is mainly concentrated around 300 nm, and 19.7% of the particles have diameters of 290–295 nm. The particle size distribution of CMIO kept for 12 months is also concentrated around 300 nm, in which particles with a size of 290–295 nm account for 18.4%. The average particle size of the newly prepared CMIO is 305.7 nm, whereas the average particle size of the CMIO kept for 12 months is 320.1 nm.

Notably, surface modifiers were not used for the C_60_ nanoparticles. However, the particle size of CMIO kept for 12 months showed no significant change. This phenomenon is due to the fact that C_60_ can dissolve in insulating oil. It should be noted that although the CMIO was stable, the observation period was limited; we will focus on this stability over a longer period. Moreover, aging is a problem faced by transformer oil, and the effect of aging on the solubility and stability of C_60_ nanoparticles in transformer oil remains to be further studied.

### 3.2. Micromorphology of Nano-C_60_

**Figure 5:**
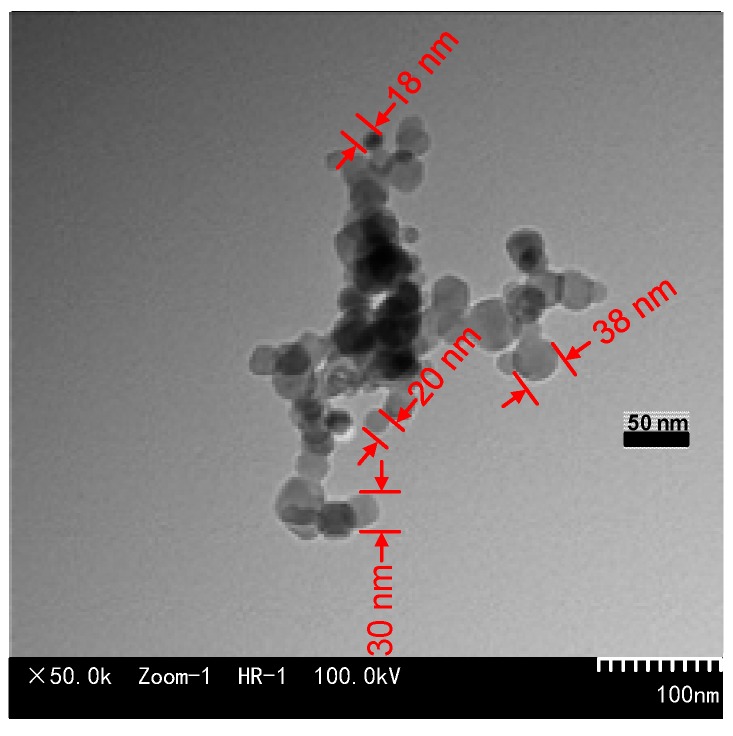
TEM image of the nano-C_60_ particles.

We suspended C_60_ particles in absolute ethyl alcohol and dripped the mixture onto an ultra-thin carbon mesh. The internal morphology of the sample on ultra-thin carbon mesh was observed by TEM after the alcohol had completely volatilized. The test results are shown in Figure 5. The C_60_ nanoparticles are round, and the diameters of most particles are less than 30 nm.

The particle sizes of the C_60_ nanoparticles suspended in CMIO increase compared with the particle sizes of the C_60_ nanoparticles in Figure 5. This increase indicates that some C_60_ nanoparticles conglomerated during the preparation process, possibly because no surface modifiers were applied to the C_60_ nanoparticles. However, CMIO without surface modifiers still shows good stability.

### 3.3. Breakdown Characteristics of FO and CMIO

**Figure 6:**
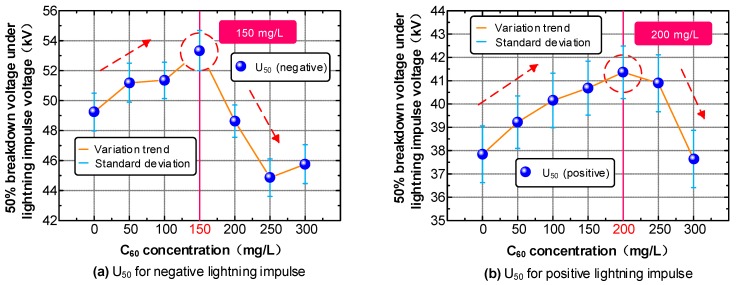
Lightning breakdown characteristics of CMIO and FO. (**a**) U_50_ for negative lightning impulse, (**b**) U_50_ for positive lightning impulse.

In this study, we used the standard lightning impulse wave of positive and negative polarity to determine the breakdown performance of FO and CMIO. The test results are plotted in Figure 6.

The C_60_ nanoparticles have a positive influence on the impulse breakdown performance of insulating oil, as can be observed in Figure 6. The positive and negative lightning impulse breakdown voltages of CMIO are simultaneously improved. This improvement in negative lightning impulse breakdown voltage was not observed from the traditional nanoparticle modified insulating oil we mentioned before. As shown in Figure 6a, the negative lightning impulse breakdown voltage tends to increase first, then decrease with increasing C_60_ concentration. However, when the concentration is 300 mg/L, the negative lightning impulse breakdown voltage has a small recovery. The breakdown voltage peaks at 150 mg/L (U_50_ = 54.23 kV). The positive lightning impulse breakdown voltage also increases first, then decreases with increasing C_60_ concentration, as shown in Figure 6b. Breakdown voltage reaches its peak value (U_50_ = 41.36 kV) at 200 mg/L C_60_ concentration. The breakdown voltage drops sharply after at 250 mg/L C_60_ concentration, and the positive lightning impulse breakdown voltage is already lower compared with that of FO at 300 mg/L.

The positive and negative lightning impulse breakdown voltage of CMIO increase by 7.51% and 8.33% at 150 mg/L relative to those of FO. The positive lightning impulse breakdown voltage of CMIO increases by 9.30%, but the negative lightning impulse breakdown voltage decreases by 1.24% at 200 mg/L C_60_ concentration. Therefore, the optimum concentration of CMIO for improving lightning impulse breakdown performance is 150 mg/L.

**Figure 7:**
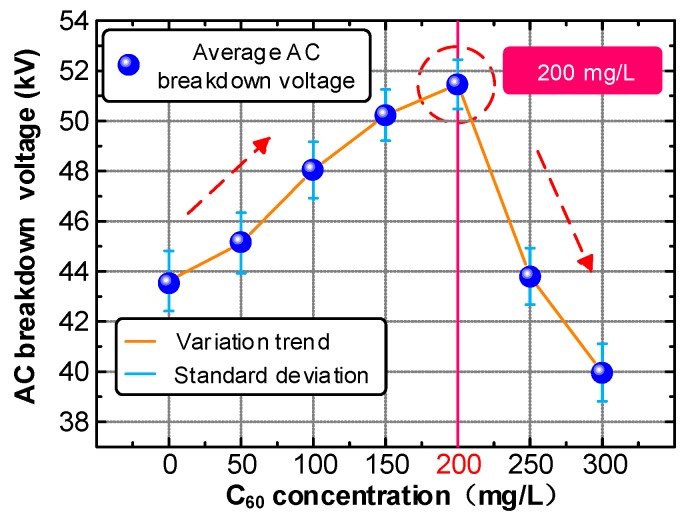
AC breakdown characteristics of CMIO and fresh oil (FO).

The average AC (50 Hz) breakdown voltage of CMIO and FO at different concentrations were further measured. The test results are shown in Figure 7.

The test results show that the average AC breakdown voltage of CMIO increases at first, then decreases with increasing C_60_ concentration. The AC average breakdown voltage reaches its peak (18.0% higher compared with FO) at 200 mg/L concentration. The average AC breakdown voltage decreases sharply at C_60_ concentrations between 200 and 250 mg/L. At 250 mg/L C_60_ concentration, the AC breakdown voltage is slightly higher than that of FO. The lowest average AC breakdown voltage is observed at 300 mg/L C_60_ concentration. Considering the results of the lightning impulse breakdown voltage test, we can conclude that the optimum modification concentration of CMIO is 150 mg/L.

### 3.4. Dielectric Characteristics of FO and CMIO

**Figure 8:**
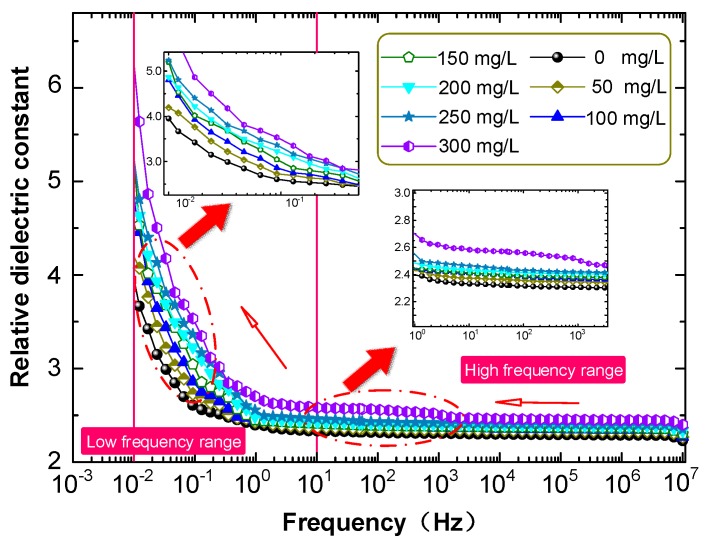
Broadband dielectric spectrum of CMIO and FO.

The influence of C_60_ nanoparticles on the dielectric properties of insulating oil was investigated by measuring the dielectric constants of CMIO samples with different concentrations at different frequencies, as shown in Figure 8.

The relative dielectric constants of FO and CMIO have minimal changes at testing frequencies ranging from 10 Hz to 10^7^ Hz. The relative dielectric constant of FO is 2.35, whereas the relative dielectric constants of CMIO (50–300 mg/L) are 2.37, 2.40, 2.41, 2.42, 2.45, and 2.57 at 50 Hz. The relative dielectric constants of CMIO and FO increase sharply with the decrease of test frequency in the range of 0.01–10 Hz. Notably, the dielectric constant of CMIO is slightly higher than that of FO across the entire testing frequency range.

Transformer insulating oil is nonpolar dielectric and its relative dielectric constant is about 2.2. The polarization equation of nonpolar liquid medium can be expressed as [36]:

(1) $$\frac{\varepsilon_{r}-1}{\varepsilon_{r}+2}=\frac{N\alpha }{3\varepsilon_{0}}$$

where *ε_r_* is the relative dielectric constant of dielectrics, *N* is the number of molecules per unit volume, *ε_0_* is the vacuum dielectric constant, and *α* is the total micro-polarizability of molecule. In nonpolar dielectrics, *α* is the electron displacement polarizability *α_e_*.

C_60_ is a nonpolar molecule, and its static dielectric constant is approximately 3.61 [22]. The high frequency dielectric constant of C_60_ is 2.6 ± 0.1 [37]. The solid–liquid system composed of C_60_ and insulating oil can be treated as nonpolar dielectric, and Equation (1) can be rewritten as:

(2) $$\frac{\varepsilon_{r}-1}{\varepsilon_{r}+2}=\frac{C_{B}\alpha_{e}}{3\varepsilon_{0}}$$

where *C_B_* is the molecule number per unit volume of dielectric, and *α_e_* is the electron displacement polarizability.

Assuming that *C_oil_* is the molecule number of oil per unit volume of CMIO and *C_C60_* is the molecule number of C_60_ per unit volume of CMIO, Equation (2) becomes:

(3) $$\frac{\varepsilon_{r}-1}{\varepsilon_{r}+2}=\frac{C_{oil}\cdot \alpha_{e1}+C_{C_{60}}\cdot \alpha_{e2}}{3\varepsilon_{0}}$$

where *α_e1_* is the electron displacement polarizability of the oil molecule, and *α_e2_* is the electron displacement polarizability of the C_60_ molecule.

The relative dielectric constant of C_60_ is larger than that of insulating oil, so *α_e2_*>*α_e1_*. The number of molecules of solids is greater than the number of molecules of liquids in the same volume. Therefore, according to Equation (3), the relative dielectric constants of all the CMIO samples are greater than dielectric constant of FO, and the relative dielectric constant of CMIO increases with concentration. This result is consistent with the test results shown in Figure 8. 

**Figure 9:**
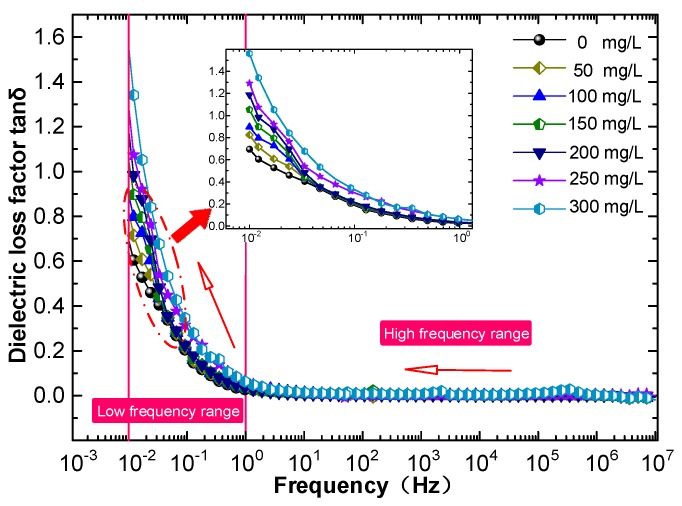
Broadband dielectric loss spectrum of CMIO and FO.

The dielectric loss factor of FO and CMIO with different concentrations at different frequencies were measured, as shown in Figure 9.

The results show that the *tanδ* of CMIO is almost the same as that of FO from 1 Hz to 10^7^ Hz. At power frequency (50Hz), the *tanδ* of FO is 0.00537, and the *tanδ* of CMIO with increasing concentrations (50–300 mg/L) is 0.00548, 0.00559, 0.00568, 0.00651, 0.00741, and 0.00843.

In the low frequency range (less than 1 Hz), the *tanδ* of CMIO is slightly higher than that of FO. The *tanδ* of all the samples increase with decreasing frequency.

The *tanδ* of dielectrics can be expressed as:

(4) $$\tan\delta =\frac{I_{p}}{I_{q}}=\frac{(\gamma +g)\cdot SE}{\omega \varepsilon_{0}\varepsilon_{r}SE}=\frac{\gamma +g}{\omega \varepsilon_{0}\varepsilon_{r}}$$

where *I_p_* is active current, *I_q_* is reactive current, *S* is cross-sectional area, *E* is the effective value of electric field strength, *γ* is dielectric conductivity, *g* is relaxation polarization equivalent conductivity, and *ω* is angular frequency.

Electron displacement polarization is the main polarization form of nonpolar and weak polar liquid dielectrics. Their dielectric loss is mainly due to conductivity loss. Relaxation polarization loss can be neglected. The dielectric loss factor in Equation (4) can be rewritten as follows:

(5) $$\tan\delta =\frac{\gamma }{\omega \varepsilon_{0}\varepsilon_{r}}$$

In nonpolar dielectric, the relative dielectric constant barely changes with frequency. The relative dielectric constant increases slightly at a low frequency (less than 1 Hz). The dielectric conductivity of insulating oil varies little with frequency, and any change is considered negligible. 

According to Equation (5), when changes in *γ* and *ε_r_* are not considered, the *tanδ* is approximately an inverse proportional function of the angular frequency *ω*, which corresponds to the trend of *tanδ* with frequency in Figure 9. 

C_60_ is a semiconductor and has greater electrical conductivity than insulating oil. Therefore, the conductivity of CMIO improves and increases with concentration. According to Equation (5), the *tanδ* of CMIO is greater than that of FO. The *tanδ* value increases with concentration, and this result is consistent with the test results.

## 4. Analysis and Discussion

### 4.1. Trap Characteristics

**Figure 10:**
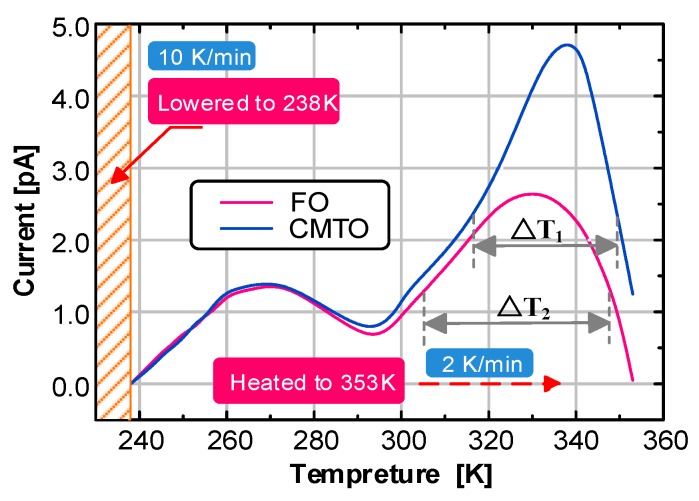
TSC test results of FO and CMIO.

The thermal stimulation currents of CMIO and FO were tested with the TSC test system. The test results are shown in Figure 10.

To obtain accurate trap parameters and fit the TSC curve and separate peaks, we used a TSC synthesis algorithm and combined relaxation and thermal excitation process methods.

First, the thermal excitation process was described by the general dynamic equation [38] shown in Equation (6):

(6) $$I(t)=-\frac{dn}{dt}={\left(\frac{n}{n_{0}}\right)}^{b}sn_{0}\exp(-\frac{E}{kT})$$

where *I* is the heat release intensity in A, which corresponds to the thermal luminescence intensity or the thermal stimulated current intensity. *n* is the carrier concentration of the trap in m^−3^, *s* is the frequency factor, *n_0_* is the initial concentration of carriers of the trap in m^−3^, *E* is the activation energy (trap level) in eV, *k* is the Boltzmann constant in eV/K, *T* is the absolute temperature in K, and *b* is the kinetic order. Usually, *b* only takes 1, 2, and 1.5 [39].

The solution of Equation (6) is:

(7) $$I(T)=n_{0}s\exp(-\frac{E}{kT})\exp(-\frac{s}{\beta }\int_{T_{0}}^{T}e^{-\frac{E}{kT{}^{\prime }}}dT{}^{\prime })(b=1)$$

(8) $$I(T)=n_{0}s\exp(-\frac{E}{kT})[1+(b-1)\frac{s}{\beta }\int_{T_{0}}^{T}e^{-\frac{E}{kT{}^{\prime }}}dT{}^{\prime }){]}^{\frac{b}{1-b}}(b\neq 1)$$

where *T_0_* is the initial temperature in K.

If a linear temperature rise process exists, that is, *T* = *T*_0_ + *βt*, then at the peak of curve *I*(*T*)

(9) $$\frac{\beta E}{kT_{m}{}^{2}}=se^{-\frac{E}{kT_{m}}}(b=1)$$

(10) $$1+\frac{(b-1)s}{\beta }\int_{T_{0}}^{T_{m}}e-\frac{E}{kT{}^{\prime }}dT{}^{\prime }=\frac{bskT_{m}{}^{2}}{\beta }e^{-\frac{E}{kT_{m}}}(b\neq 1)$$

where *T_m_* is the temperature corresponding to the peak current in K.

After calculating the logarithm of the two sides of Equation (6), we obtained the following equation:

(11) $$\ln I(t)=b\ln(\frac{n}{n_{0}})+\ln(sn_{0})-\frac{E}{kT}$$

After points (*T*_1_, *I*_1_) and (*T*_2_, *I*_2_) on the TSC curve were substituted into Equation (11),

(12) $$\ln(\frac{I_{2}}{I_{1}})=b\ln(\frac{n_{2}}{n_{1}})-\frac{E}{k}(\frac{1}{T_{2}}-\frac{1}{T_{1}})$$

Then, using the two points (*T*_3_, *I*_3_) and (*T*_1_, *I*_1_) on the TSC curve, we obtained:

(13) $$\ln(\frac{I_{3}}{I_{1}})=b\ln(\frac{n_{3}}{n_{1}})-\frac{E}{k}(\frac{1}{T_{3}}-\frac{1}{T_{1}})$$

After Equations (12) and (13) were combined, the activation energy (trap level) and kinetic progression were calculated by:

(14) $$E=\frac{k[\ln(\frac{I_{3}}{I_{1}})\ln\frac{n_{2}}{n_{1}}-\ln(\frac{I_{2}}{I_{1}})\ln\frac{n_{3}}{n_{1}}]}{\left[(\frac{1}{T_{2}}-\frac{1}{T_{1}})\ln(\frac{n_{3}}{n_{1}})-(\frac{1}{T_{3}}-\frac{1}{T_{1}})\ln(\frac{n_{2}}{n_{1}})\right]}$$

(15) $$b=\left[\ln(\frac{I_{2}}{I_{1}})+\frac{E}{k}(\frac{1}{T_{2}}-\frac{1}{T_{1}})\right]/\ln(\frac{n_{2}}{n_{1}})$$

In simple TSC curves (isolated peak), provided that three points (*I*_1_, *T*_1_), (*I*_2_, *T*_2_), and (*I*_3_, *T*_3_) are selected on the curves, *E* and *b* can be calculated according to Equations (14) and (15), and *s* can be calculated from Equations (9) and (10). Meanwhile, *n* can be calculated by:

(16) $$n=\frac{1}{\beta }\int_{T}^{T\infty }IdT{}^{\prime }$$

where *T*_∞_ is the temperature at which the TSC curve decays to zero (actually below the sensitivity of the meter).

For the calculation of $\int_{T_{0}}^{T_{m}}e^{-\frac{E}{kT{}^{\prime }}}dT{}^{\prime }$ in Equation (10), the Gaussian quadrature formula can be used. For the $\int_{T}^{T\infty }IdT{}^{\prime }$ in Equation (16), the following method can be used. First, a point is taken every 1 K on the TSC curve, and the curve is discretized into *N* points. Then, by using the *N* points as interpolation points, cubic spline interpolation is carried out and the TSC curve is expressed by piecewise cubic spline function, so the integral in Equation (16) can be transformed into the integral of cubic spline function [40]. 

However, in actual experimental conditions and results, completely applying the algorithm is difficult. The current of most samples in the actual measurement were attenuated to zero before the end of the TSC measurement given the limitation of the temperature tolerance of the heater. Therefore, we failed to calculate *n* by using Equation (16). 

Another drawback of the thermal excitation process is that the trap level *E* calculated from the multipoint values on the actual curve vary greatly in some cases. Therefore, we had to use the relaxation process method to obtain the preliminary theoretical curve of the measured TSC.

The activation energy (trap level) *E* can be calculated by the half-width formula [41]:

(17) $$E=\frac{2.47T_{m}{}^{2}k}{\Delta T}$$

where Δ*T* is the temperature difference corresponding to the half-peak in K, and *k* is the Boltzmann constant in eV/K.

Assuming that the measured TSC curve is a single frequency relaxation process, according to Debye theory, the short circuit TSC can be expressed as [28]:

(18) $$I=A\exp[-\frac{E}{kT}-\frac{1}{\tau_{0}\beta }\int_{T_{0}}^{T}\exp(-\frac{E}{kT})dT]$$

where *τ_0_* is the time constant, and *β* is the heating rate in K/min.

The relationship between *I* and *T* can be calculated by using Equation (18) combined with the TSC curve.

Using the Gorbachev approximation,

(19) $$\begin{array}{l} \int_{T_{0}}^{T}\exp(-\frac{E}{kT})dT=\int_{0}^{T}\exp(-\frac{E}{kT})dT-\int_{0}^{T_{0}}\exp(-\frac{E}{kT})dT\\ =\int_{0}^{T}\exp(-\frac{E}{kT})dT+C\approx (\frac{kT^{2}}{E+kT})\exp(-\frac{E}{kT})+C \end{array}$$

*C* in Equation (19) is a constant independent of *T*, so

(20) $$I\approx A\exp[-\frac{E}{kT}-\frac{1}{\tau_{0}\beta }(\frac{kT^{2}}{2kT+E})\exp(-\frac{E}{kT})-\frac{C}{\tau_{0}\beta }]$$

Make $\frac{dI}{dT}=0$, get

(21) $$\tau_{0}\beta \approx \frac{kT^{2}}{E}[\frac{E}{E+2kT}\exp(-\frac{E}{kT})+\frac{2kT}{E+2kT}\exp(-\frac{E}{kT})]=\frac{kT^{2}}{E}\exp(-\frac{E}{kT})$$

$\frac{dI}{dT}=0$ corresponds to *I*_m_, *T*_m_ in the TSC spectrum, substituting *I*_m_ and *T*_m_ into Equation (21).

(22) $$\tau_{0}\beta \approx \frac{kT_{m}{}^{2}}{E}\exp(-\frac{E}{kT_{m}})$$

From Equation (20), we can obtain the following equation:

(23) $$\begin{array}{l} I_{m}\approx A\exp(-\frac{C}{\tau_{0}\beta })\exp[-\frac{E}{kT_{m}}-\frac{1}{\tau_{0}\beta }(\frac{kT_{m}{}^{2}}{2kT_{m}+E})\exp(-\frac{E}{kT_{m}})]\\ =A{}^{\prime }\exp[-\frac{E}{kT_{m}}-\frac{1}{\tau_{0}\beta }(\frac{kT_{m}{}^{2}}{2kT_{m}+E})\exp(-\frac{E}{kT_{m}})] \end{array}$$

By substituting *I*_m_ and *T*_m_ into Equation (23), the value of *A'* can be obtained. Then, *A'* and Equations (20), (22), and (23) can be used in the determination of the fitting curve of *I*-*T* (TSC curve).

In the relaxation process method, the TSC curve is assumed to be a single relaxation process, and some approximations are made. Therefore, the theoretical curve obtained by this method does not fit the measured curve well but can be used as the basis of the thermal excitation process method.

We substituted the data points selected from the theoretical curve of the relaxation process algorithm into the thermal excitation process algorithm to calculate the corresponding trap parameters. Then, we compared the theoretical curves obtained from these trap parameters with the measured TSC curves and gradually adjusted the trap depth and other trap parameters until the calculated fitting curves were the same as the measured curves. The algorithm which combines the relaxation process method with the thermal excitation process method was called the synthesis algorithm.

**Figure 11:**
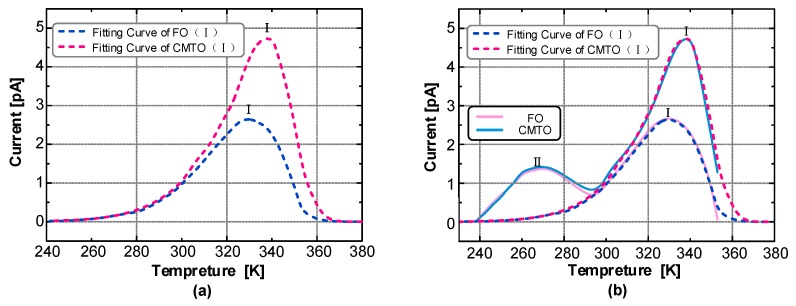
(**a**) Peak I fitting curve of FO and CMIO, (**b**) Comparison of Peak I fitting and measured curves.

The main peak I was fitted first, which is on the right side of the TSC curve. The fitted and actual curves are shown in Figure 11. 

That the TSC curve fitted by the steps has a high degree of coincidence with the actual curve can be seen from Figure 11b. Despite the slight deviation between the fitting and actual curves in the initial and end parts, the remaining parts basically coincide.

**Figure 12:**
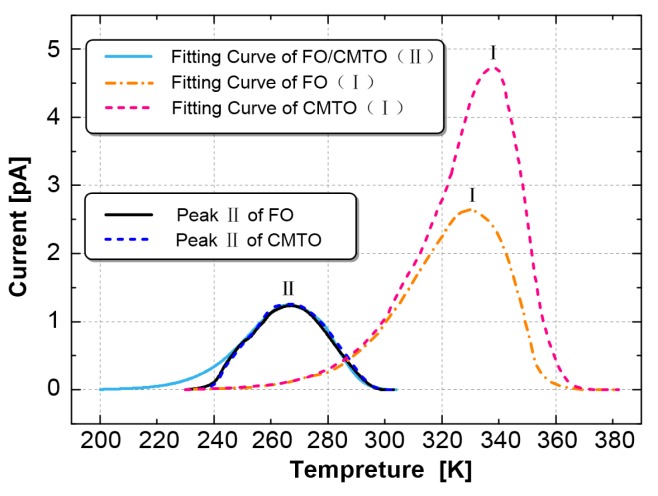
Measured curve of peak II of FO and CMIO and fitting curve of peaks I and II.

The measured TSC curve has two peaks, main peak I and secondary peak II. Peak separation is needed to complete the fitting of the entire TSC curve. The curve measured in the experiment is actually the superposition of main peak I and secondary peak II. After the fitting of main peak I was completed, the measured curve of peak II was obtained by subtracting the fitting curve of main peak I from the measured TSC curve. Then, the synthesis algorithm was used to fit secondary peak II. Finally, we obtained the fitting of the whole measured TSC curve, as shown in Figure 12.

As shown in Figure 11 and Figure 12, the curve fitted by the synthetic algorithm has a high overall coincidence with the measured curve, but the curves differ in the initial rising and final falling parts. Moreover, this method can complete the TSC curve fitting including multiple peaks.

Given that the temperature is approximately proportional to time, the integration of the current with temperature can be used in the calculation of the amount of charge released by the trap [41], as shown in the following equation:

(24) $$Q=\int_{t_{0}}^{t_{1}}I(t)dt=\frac{1}{\delta }\int_{T_{0}}^{T_{1}}I(T)dT$$

where *Q* is total charge of the trap in C, *δ* is the heating rate in K/min, *T*_0_ is the experimental starting temperature, and *T*_1_ is the temperature at which the current drops to zero.

The characteristic values of the TSC trap parameter of the insulating oil can be obtained by fitting and calculating, as shown in Table 4.

The peak II trap parameters of FO and CMIO are almost the same. All the traps are shallow with levels of about 0.42 eV. Nano-C_60_ particles do not change the trap characteristics of CMIO at peak II but mainly affect peak I. At peak I, the peak current of CMIO (4.84 pA) is 1.81 times that of FO (2.68 pA), the trap level of CMIO (0.718 eV) is 1.42 times that of FO (0.505 eV), and the trap charge of CMIO (5.12 nC) is 1.41 times that of FO (3.63 nC). Given that the peak value of the TSC curve is related to maximum trap density in a dielectric liquid [28], CMIO is believed to have a higher trap density than FO. Therefore, we infer that the addition of nano-C_60_ particles increases the trap energy level depth and trap density of insulating oil.

### 4.2. Adsorption of Charge Carriers and Percolation Threshold Phenomenon

Electrical conductivity in an insulation material depends on the amount or concentration of charge carriers (*N_i_*), the charge of charge carriers (*q_i_*), and their mobility (*µ_i_*), which can be expressed as [42]:

(25) $$\sigma =\sum_{i}N_{i}q_{i}\mu_{i}$$

Decreasing the concentration of potential charge carriers by increasing the chemical cleanliness of the insulating material, and reducing their mobility by introducing a filler that contains traps for charge carriers are equally important to the reduction of conductivity [42].

**Figure 13:**
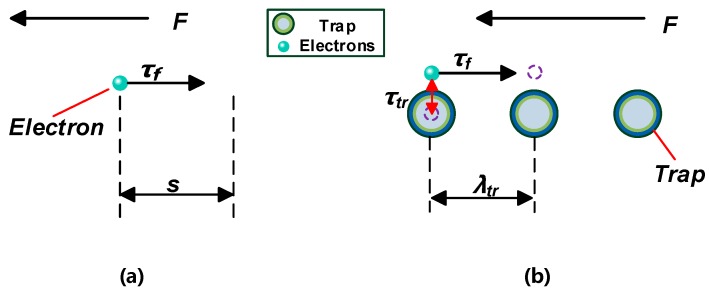
(**a**) Carrier migration in the medium (without traps), (**b**) carrier migration in the medium (with traps).

The trap fill effect is not needed at small trap charge [43]. The carrier mobility is shown in Figure 13.

According to the formula for calculating the mean free path of carriers *λ_f_* [44]:

(26) $$\lambda_{f}=\mu_{0}F\cdot \tau_{F}$$

where *μ_0_* is the carrier mobility when no trap exists in the dielectric material, *F* is the external electric field, and *τ_F_* is the lifetime of the carrier. 

As shown in Figure 13a, when no trap exists in the dielectric material under the action of electric field *F*, the time *τ_f_* required for the *s* distance of carrier migration can be calculated as follows:

(27) $$\tau_{f}=\frac{s}{F\cdot \mu_{0}}$$

When traps exist in dielectric materials (Figure 13b), carriers are trapped. If the carrier resides in the trap for a time of *τ_tr_*, the total time *τ_to_* spent for carrier migration in the *s* distance is:

(28) $$\tau_{to}=\tau_{f}+\tau_{tr}$$

The effective mobility of carriers can be calculated by [45]:

(29) $$\mu_{e}{}_{f}=\frac{\lambda_{tr}}{F\cdot \tau_{to}}=\frac{\mu_{0}\cdot \tau_{f}}{\tau_{f}+\tau_{tr}}$$

Generally, the carrier residence time *τ_tr_* in the shallow trap is very short and is shorter than the carrier lifetime *τ_f_*. According to Equation (29), the carrier effective mobility *μ_ef_* is approximately equal to the carrier mobility *μ_0_* in the shallow trap. Therefore, after being trapped by shallow traps in the FO, electrons can easily escape again. The frequency of electrons entering the trap is equivalent to the frequency of electrons leaving the trap, and the trapping ability of the shallow trap is weak.

Carrier trapping time in deep traps is generally longer than carrier lifetime [46]. Deep traps form a charge trapping center, which causes space charge accumulation in the dielectric. At this time, carrier effective mobility *μ_ef_* is smaller than carrier mobility *μ_0_*.

CMIO has a deeper trap level and a higher trap density than FO, and the frequency of electrons entering the trap is higher than the frequency of electrons leaving the trap. Moreover, the binding ability of electron trap is strong. Carrier mobility in CMIO is lower than that in FO. Therefore, the number of electrons trapped by the deep and dense traps in CMIO increases during discharge development, and this increase weakens the development of streamers and increases breakdown voltage.

The volume fraction of a conducting filler in an insulating matrix has a critical level, above which the electrical or thermal conductivity increases dramatically due to the development of a continuous path of electron transport [47]. This critical volume fraction, which is the minimum concentration required to create an infinitely connected network, is called the percolation threshold [48]. The resistivity or conductivity near the percolation threshold can then be described by Equation (30) [47,49]:

(30) $$\sigma \propto {\left(\upsilon -\upsilon_{c}\right)}^{t}$$

where *υ* is the volume fraction of filler, *υ*_c_ is the percolation threshold, and *t* is a power law constant that depends on the geometry of the system.

For the reduction of the charge mobility or DC conductivity in composites based on non-insulating particles, the particle volume fraction in the composite system should typically be lower than the percolation threshold, where conduction may occur by tunneling between particles because of short interparticle distance [50,51]. At this critical interparticle distance, the trapping of charge carriers by non-insulating particles is not beneficial for conductivity reduction.

**Figure 14:**
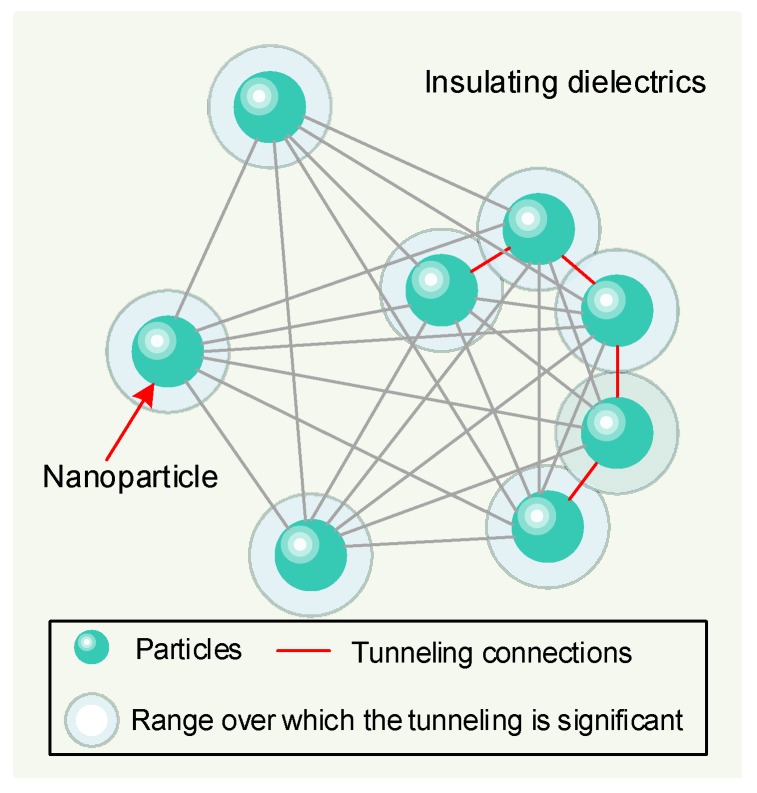
Schematic diagram of tunneling effect.

The tunneling effect can be described as shown in Figure 14.

When the distance between particles is less than the critical interparticle distance (the red line in Figure 14), a system of geometrically disconnected particles occurs. In this system, an insulating layer separates adjacent particles and electrically these are all connected by tunneling [49].

In CMIO, the volume fraction of C_60_ nanoparticles must not exceed the percolation threshold. As observed in our experimental data, the breakdown voltage of CMIO drops sharply at a certain C_60_ concentration. This sharp drop may be related to the percolation threshold.

### 4.3. Relaxation Time Constant and Electrical Potential Distribution of C_60_ Nanoparticles

The conductivity of C_60_ single crystal at room temperature is approximately 1.7 × 10^−6^ S/m [52]. The relaxation time constant of C_60_ nanoparticles in CMIO can be calculated by the following equation [53]:

(31) $$\tau_{r}=\frac{2\varepsilon_{1}+\varepsilon_{2}}{2\sigma_{1}+\sigma_{2}}$$

where *ε_1_* is the relative permittivity of insulating oil (2.2*ε_0_*), *ε_2_* is the relative permittivity of C_60_ nanoparticles (4*ε_0_*), σ_1_ is the conductivity of insulating oil (10^−12^ S/m), σ_2_ is the conductivity of C_60_ nanoparticles (1.7 × 10^−6^ S/m), and *ε_0_* is the vacuum permittivity (8.85 × 10^−12^ F/m).

The relaxation time constant of the C_60_ nanoparticles in CMIO calculated from Equation (31) is 4.37 × 10^−5^ s. According to the fast electron capture theory [53], the relaxation time constant *τ_r_* of nanoparticles represents the speed of polarization process of nanoparticles. However, the relaxation time constant of C_60_ nanoparticles in CMIO is much larger than the development time of the streamer (the development time range of streamer is from several hundred nanoseconds to several microseconds). Therefore, C_60_ nanoparticles have other mechanisms which act on the modification of insulating oil. 

Transformer insulating oil is a nonpolar dielectric. Few dipoles are present in the filtered and vacuum dried oil samples. However, due to the difference in relative dielectric constant between C_60_ and insulating oil, nanoparticles generate induced dipole moments under a high electric field [54]. The surface of the nanoparticles will have a charge distribution. The charge distributed on these surfaces will trap the rapidly migrating electrons and hinder the development of streamers.

**Figure 15:**
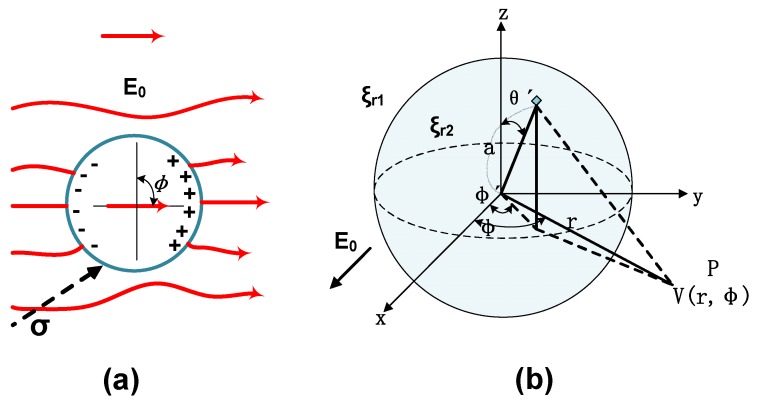
(**a**) Dipole electric charge and electric line of force distributions near spherical dielectric induced by an electric field E_0_, (**b**) The spherical dielectric is present in the base dielectric material.

Assume that C_60_ nanoparticles in insulating oil is a standard spherical dielectric. The spherical dielectric (diameter 2a, relative permittivity ε_2_) is present in the base dielectric material (insulating oil, relative permittivity ε_1_), as shown in Figure 15b. Figure 15a is dipole electric charge and the electric line of force distributions near the spherical dielectric induced by an electric field *E_0_*.

The surface charge density *σ_p_* and electrical potential *V(r, Φ)* of the spherical dielectric can be calculated by using Equations (32) and (33):

(32) $$\sigma_{p}=\varepsilon_{0}E_{0}\left(1-\frac{3\varepsilon_{r1}}{2\varepsilon_{r1}+\varepsilon_{r2}}\right)\cos\phi^{\prime }\sin\theta^{\prime }$$

(33) $$\frac{V\left(r,\phi \right)}{aE_{0}}=\frac{1}{4\pi }\left[1-\frac{3\varepsilon_{r1}}{2\varepsilon_{r1}+\varepsilon_{r2}}\right]\times \int_{-\pi }^{+\pi }\int_{0}^{+\pi }\frac{\sin^{2}\theta^{\prime }d\theta^{\prime }\cos\phi^{\prime }d\phi^{\prime }}{\sqrt{1+{\left(r/a\right)}^{2}-2\left(r/a\right)\sin\theta^{\prime }\cos\left(\phi -\phi^{\prime }\right)}}$$

**Figure 16:**
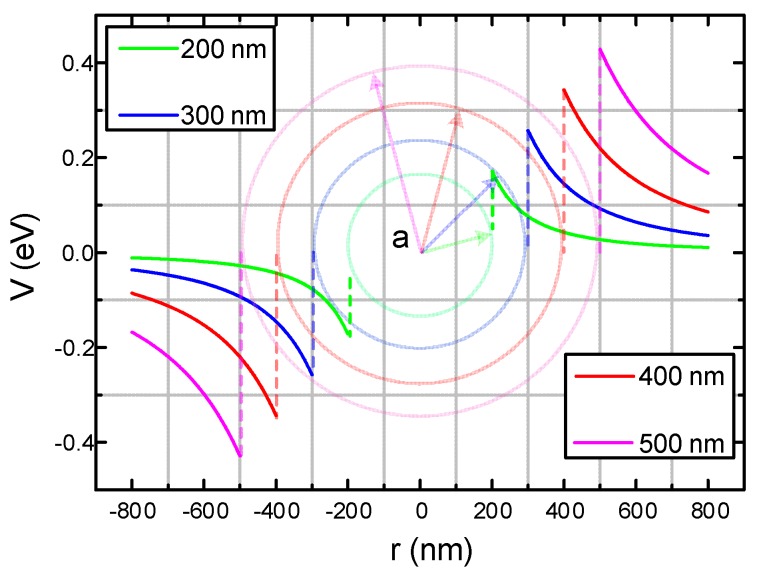
Electrical potential of C_60_ spherical nanoparticles along the electric field *E_0_* and central axis.

According to the TSC test procedure in Section 4.1 Trap Characteristics, the electric field strength *E_0_*=4 kV/mm applied in the experiment, the relationship between the particle size of C_60_ nanoparticles in CMIO and the electrical potential along the electric field and the central axis can be obtained by using Equations (32) and (33), as shown in Figure 16. 

As shown in Figure 16, under *E_0_* of 4 kV/mm, the surface charge potential of a C_60_ nanoparticle is 0.257 eV when the particle size is 300 nm. The TSC test result shows that the maximum trap level generated by C_60_ nanoparticles in CMIO is 0.718 eV. When the error of the model itself is excluded, the surface electric potential calculated according to this model is smaller than the trap value actually measured in the TSC test. This difference may be attributed to the following reasons:

- In this model, the applied electric field *E_0_* is uniform. In the TSC test, the small shim in the oil tank (Figure 3) affects the uniformity of the electric field distribution.
- The nanoparticles (or nanoparticle agglomerates) are assumed to be standard spheres in this model, and thus their morphology may be different from that of C_60_ nanoparticles present in CMIO.
- More importantly, we believe that the adsorption of carriers by C_60_ in CMIO is not only the effect of the electric potential wells produced by the induced dipole consisting of C_60_ nanoparticles under a high electric field, but also the electronegativity effect of C_60_. We will further discuss the effect of C_60_ electronegativity on electron adsorption in Section 4.4.

### 4.4. Electronegativity of C_60_

C_60_ nanoparticles have strong “electronegativity” (the ability to capture electrons) and their electronic affinity *EA* can be expressed by the following equation [18]:

(34) $$EA=E(R_{e})-E^{-}(R^{-}{}_{e})$$

where *EA* is the electron affinity, *E(R_e_)* is the total energy of a stable neutral molecule, and *E^−^(R^−^_e_)* is the total energy of a stable molecule after capturing an electron.

The most accurate electron affinity for C_60_ is [17]:

(35) EA=2.683±0.008 eV

**Figure 17:**
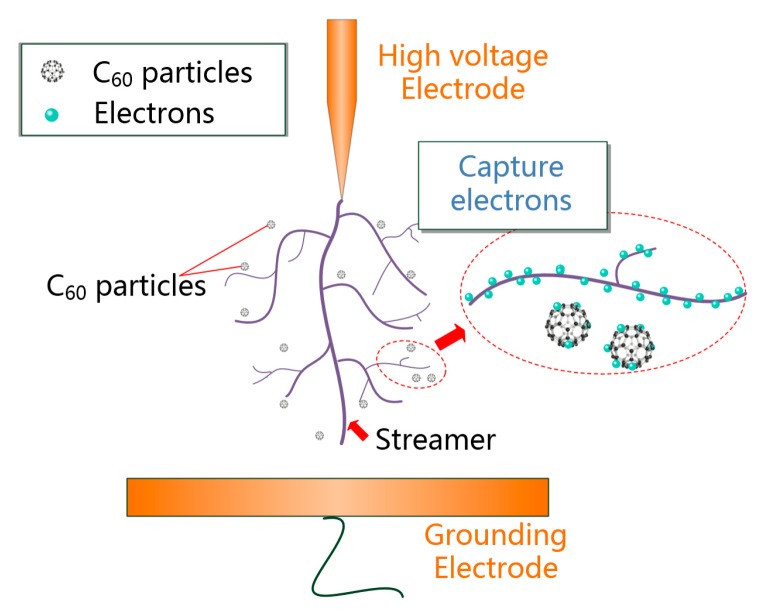
C_60_ particles capture electrons involved in streamer formation.

The *EA* of C_60_ is higher than the electronic affinity (about 1.06 eV) of SF_6,_ which is an excellent gas insulation material and has been widely used. Thus, C_60_ has a strong electron adsorption ability. The capturing effect of C_60_ added to insulating oil on electrons is shown in Figure 17.

C_60_ nanoparticles can capture electrons which reduces the number of electrons that can participate in the formation of electron avalanches under the action of electric field, thus weakening the development of streamers and increasing the breakdown voltage of CMIO.

The ability of C_60_ to absorb electrons is caused by its special structure at the microlevel. We suspect that the electron adsorption capacity resulting from the microstructure leads to the deepening of the trap shown in Section 4.1.

### 4.5. Absorption of Photons by C_60_

**Figure 18:**
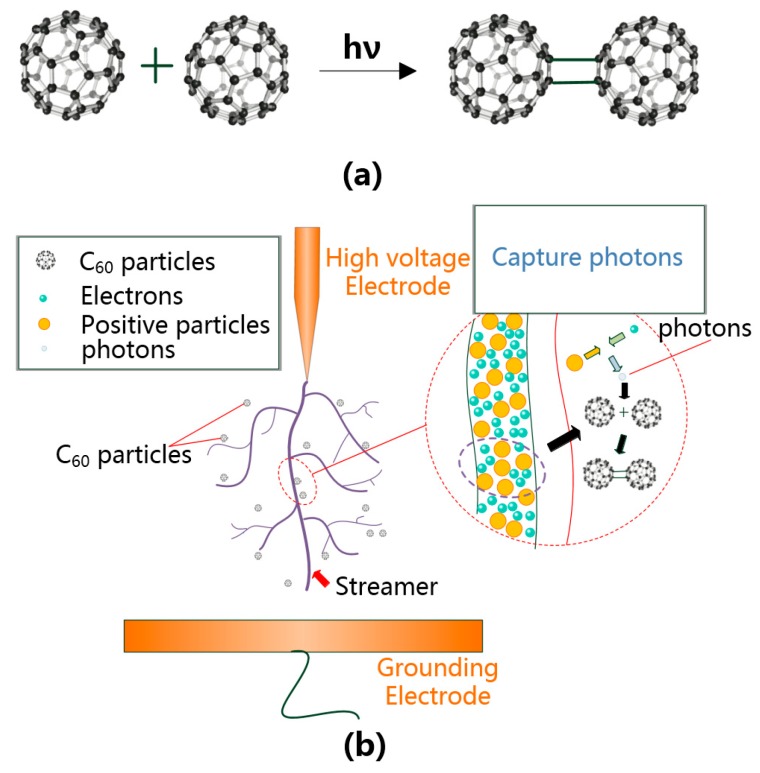
(**a**) Two C_60_ molecules absorb photons and form dimer, (**b**) C_60_ absorbs photons in the streamer through photopolymerization reaction.

The photopolymerization of C_60_ occurs under the irradiation of ultraviolet light or visible light of a specific wavelength. Irradiation results in the polymerization of two adjacent C_60_ molecules and consumption of photons [20]. The C_60_ dimer is the most abundant and simple structure in the polymer. The formation process can be explained as shown in Figure 18a.

As shown in Figure 18b, in the process of streamer development, the positive and negative ions in the electron avalanche recombine violently and emit a large number of photons. These high-energy photons cause subsequent photoionization, thus generating new free electrons (photoelectrons). C_60_ molecules can absorb photons generated during streamer development through photon absorption polymerization (most are dimers), which weakens the process of photoionization. Thus, the molecules weaken the development of streamers and improve the breakdown performance of CMIO.

We speculate that C_60_ can improve the negative impulse breakdown voltage of insulating oil because C_60_ can absorb photons. An explanation for the negative impulse breakdown voltage drop of nano-insulating oil is as follows. A large number of negatively charged slow nanoparticles remain near the negative electrode. This phenomenon weakens the electric field strength at the needle electrode and strengthens it at the plate electrode. Therefore, the enhancement of voltage is conducive to the initiation and development of streamers to the anode and decreases negative breakdown voltages with the suspension of nanoparticles [6]. 

According to this explanation, the negative impulse breakdown voltage of CMIO should be lower compared with that of FO. However, unlike other nanoparticles, the C_60_ in CMIO can absorb photons, weakens the process of photoionization, and disrupts the development of streamers. Therefore, we believe that the absorption of photons by C_60_ is the reason that C_60_ can increase the negative impulse breakdown voltage of CMIO, and this factor is what makes CMIO markedly different from other nano-insulating oils.

### 4.6. Summary of the Mechanism of C_60_

**Figure 19:**
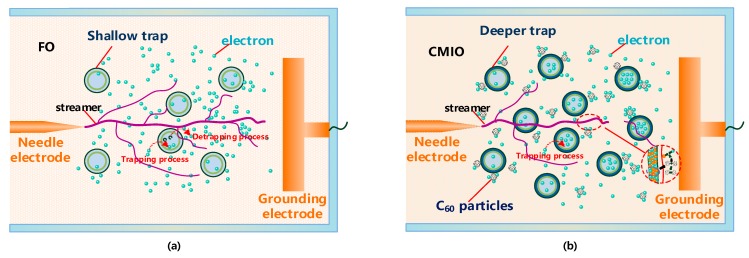
(**a**) Electron motion in FO under electric field, (**b**) Electron motion in CMIO under electric field.

In the development of discharge in FO and CMIO, the motion of electrons can be represented by Figure 19a,b. The trap parameters of insulating oil, and the electron adsorption capacity of C_60__,_ and its ability to absorb photons, affect the breakdown performance of nano-insulating oil and the development process of discharging.

As shown in Figure 19, the modification mechanism of C_60_ to improve the breakdown performance of insulating oil can be summarized by three aspects:

- The addition of C_60_ nanoparticles deepens the trap energy level and increases the trap density in insulating oil. In CMIO, the frequency of electrons entering the trap is higher than the frequency of electrons leaving the trap, and the capture ability of the electron trap is strong. Carrier mobility in CMIO is lower than that in FO. The number of electrons trapped by the deep and dense traps increases.
- C_60_ nanoparticles have a strong ability to absorb electrons. As C_60_ captures electrons, it reduces the number of electrons that can participate in the formation of electron avalanches under an electric field, thus weakening the development of streamers and increasing the breakdown voltage of CMIO.
- C_60_ molecules can absorb photons generated in the process of streamer development though photon absorption polymerization (most are dimers), which weakens the process of photoionization, and thus disrupts the development of streamers and improves the breakdown performance of CMIO. We believe that the absorption of photons by C_60_ is the reason for the improvement in the negative impulse breakdown performance of CMIO.

C_60_ can improve the breakdown performance of insulating oil while hardly increasing dielectric loss. The slight increase in CMIO relative dielectric constant is also beneficial to the insulation coordination of the transformer oil-paper insulation system. However, it should also be noted that due to the cost of C_60_ materials, the cost of using power transformers may be further increased. Although C_60_ is not toxic to the human body, whether nano-sized C_60_ in insulating oil causes environmental damage must be clarified.

## 5. Conclusions

Based on the test and analysis results, the following conclusions are reached:

The C_60_ nanoparticles are round, and their diameters are about 30 nm. The average particle size of the newly prepared CMIO is 305.7 nm, and the average particle size of the CMIO kept for 12 months is 320.1 nm. Without the use of any surface modifiers, the particle size of CMIO kept for 12 months did not change significantly. Meanwhile, the average zeta potential (pH=7, 100 mg/L) of CMIO is −41.3 mV. Therefore, the dispersion stability of CMIO is good. CMIO has better stability compared with traditional nano-modified insulating oil, especially without the use of surface modifiers for nanoparticles. 

The positive lightning impulse breakdown voltage of CMIO is not as high as that of some traditional nanoparticles (such as Fe_3_O_4_ and TiO_2_), but the negative lightning impulse breakdown voltage of CMIO is increased. This finding is good news for the engineering application of nano-modified insulating oil. According to our test results, the optimum concentration for improving the comprehensive breakdown performance of CMIO is 150 mg/L.

The dielectric constant of CMIO is slightly higher than that of FO across the entire test frequency range. In the low frequency range (less than 1 Hz), the dielectric loss factor of CMIO is slightly higher than that of FO. The dielectric loss factors of all the samples increase with decreasing frequency. According to the TSC measured curve, the peak separation, and fitting calculation results, the peak current of CMIO (4.84 pA) is 1.81 times that of FO (2.68 pA), the trap level of CMIO (0.718 eV) is 1.42 times that of FO (0.505 eV), and the trap charge of CMIO (5.12 nC) is 1.41 times that of FO (3.63 nC) at peak I. We believe that a substantial number of electrons are trapped by the deep and dense traps in CMIO, and thus the development of streamers is disrupted and breakdown voltage increases.

The breakdown performance of CMIO is higher than that of FO because of the changes in the trap parameters, the strong electron capture ability of C_60__,_ and its absorption capacity for photons. We believe that the inhibition of C_60_ nanoparticles on the photoionization process is the reason for the improvement in the negative impulse breakdown performance of CMIO.

## Figures and Tables

**Table 1:** Reduction of negative lightning impulse breakdown voltage of nano-insulating oil relative to host oil reported in the literature.

NP ^1^/Oil System	Nanoparticle Type	Concentration	Size of NP	Percentage Increase	Ref
Fe_3_O_4_/U-60 ^2^	Conductor	NM ^6^	<10 nm	−9.41%	[5]
Fe_3_O_4_/Nytro ^3^	Conductor	NM	<10 nm	−2.26%	[5]
Fe_3_O_4_/#25MO ^4^	Conductor	0.03 g/L	20 nm	−15.82%	[6]
TiO_2_/#25MO	Semiconductor	0.01 g/L	20 nm	−3.39%	[6]
Al_2_O_3_/#25MO	Insulator	0.02 g/L	20 nm	−2.53%	[6]
(Fe_3_O_4_/ MO) Aged ^5^	Conductor	NM	<10 nm	−7.75%	[7]
TiO_2_/#25MO	Semiconductor	5% *w*/*v*	<50 nm	−4.49%	[8]
Al_2_O_3_/#25MO	Insulator	5% *w*/*v*	<50 nm	−7.34%	[8]
Fe_3_O_4_/#25MO	Conductor	5% *w*/*v*	<50 nm	−6.12%	[8]
Al_2_O_3_/#25MO	Insulator	20% *w*/*v*	NM	−9.98%	[9]
TiO_2_/#25MO	Semiconductor	0.075% *v*/*v*	<10 nm	−6.84%	[10]
SiO_2_/#25MO	Insulator	20% *w*/*v*	NM	−5.93%	[11]
ZnO/#25MO	Semiconductor	0.0475% *v*/*v*	5~20 nm	−34.4%	[12]
Al_2_O_3_/#25MO	Insulator	0.1425% *v*/*v*	5~20 nm	−13.0%	[12]

^1^ Nanoparticle; ^2^ Univolt 60 Exxon mineral oil; ^3^ Nytro 10X mineral oil; ^4^ #25 mineral oil; ^5^ Compared with aged MO; ^6^ Not mentioned.

**Table 2:** Electrical properties of magnetic fluids compared with those of pure insulating oil.

Volume Fraction (%)	Loss Factor	Growth Multiple of Loss Factor	Dielectric Strength (kV)
0.0	0.00366	1	70
0.800	2.389	653	-
0.016	0.399	109	79
0.0080	0.1868	51	72
0.0040	0.1274	35	70

**Table 3:** Zeta potential of nano-C_60_ modified insulating oil (CMIO) (100 mg/L, pH=7).

**Measurement times**	**1**	**2**	**3**	**4**	**5**	**6**	**Average value/ mV**
**Zeta potential/mV**	−43.0	−40.6	−41.5	−39.7	−38.3	−45.0	−41.3
**Measurement times**	**7**	**8**	**9**	**10**	**11**	**12**	**Standard deviation**
**Zeta potential/mV**	−45.5	−41.1	−41.9	−39.7	−38.9	−41.0	2.241

**Table 4:** Characteristics of TSC trap parameters of insulating oil.

Trap Parameter	FO	CMIO
Peak Current (I)/pA	2.68	4.84
Peak Current (II)/pA	1.25	1.25
Trap level (I)/eV	0.505	0.718
Trap level (II)/eV	0.426	0.426
Q (I)/nC	3.63	5.12
Q (II)/nC	1.30	1.30
